# Deep Learning Technique for Automatic Segmentation of Proximal Hip Musculoskeletal Tissues From CT Scan Images: A MrOS Study

**DOI:** 10.1002/jcsm.13728

**Published:** 2025-02-28

**Authors:** Mahdi Imani, Jared Buratto, Thang Dao, Erik Meijering, Sara Vogrin, Timothy C. Y. Kwok, Eric S. Orwoll, Peggy M. Cawthon, Gustavo Duque

**Affiliations:** ^1^ Australian Institute for Musculoskeletal Science (AIMSS) The University of Melbourne and Western Health St. Albans Victoria Australia; ^2^ Department of Medicine‐Western Health The University of Melbourne St. Albans Victoria Australia; ^3^ School of Computer Science and Engineering The University of New South Wales Kensington New South Wales Australia; ^4^ Jockey Club Centre for Osteoporosis Care and Control, School of Public Health The Chinese University of Hong Kong Shatin Hong Kong; ^5^ Division of Endocrinology, Diabetes and Clinical Nutrition, School of Medicine Oregon Health & Science University Portland Oregon USA; ^6^ Research Institute California Pacific Medical Center San Francisco California USA; ^7^ School of Medicine University of California San Francisco San Francisco California USA; ^8^ Bone, Muscle & Geroscience Group Research Institute of the McGill University Health Centre Montreal Quebec Canada; ^9^ Dr. Joseph Kaufmann Chair in Geriatric Medicine, Department of Medicine McGill University Montreal Quebec Canada

**Keywords:** artificial intelligence, CT scan, image segmentation, intermuscular adipose tissue, marrow adipose tissue, osteoporosis, sarcopenia

## Abstract

**Background:**

Age‐related conditions, such as osteoporosis and sarcopenia, alongside chronic diseases, can result in significant musculoskeletal tissue loss. This impacts individuals' quality of life and increases risk of falls and fractures. Computed tomography (CT) has been widely used for assessing musculoskeletal tissues. Although automatic techniques have been investigated for segmenting tissues in the abdomen and mid‐thigh regions, studies in proximal hip remain limited. This study aims to develop a deep learning technique for segmentation and quantification of musculoskeletal tissues in CT scans of proximal hip.

**Methods:**

We examined 300 participants (men, 73 ± 6 years) from two cohorts of the Osteoporotic Fractures in Men Study (MrOS). We manually segmented cortical bone, trabecular bone, marrow adipose tissue (MAT), haematopoietic bone marrow (HBM), muscle, intermuscular adipose tissue (IMAT) and subcutaneous adipose tissue (SAT) from CT scan images at the proximal hip level. Using these data, we trained a U‐Net–like deep learning model for automatic segmentation. The association between model‐generated quantitative results and outcome variables such as grip strength, chair sit‐to‐stand time, walking speed, femoral neck and spine bone mineral density (BMD), and total lean mass was calculated.

**Results:**

An average Dice similarity coefficient (DSC) above 90% was observed across all tissue types in the test dataset. Grip strength showed positive correlations with cortical bone area (coefficient: 0.95, 95% confidence interval: [0.10, 1.80]), muscle area (0.41, [0.19, 0.64]) and average Hounsfield unit for muscle adjusted for height squared (AHU/h^2^) (1.1, [0.53, 1.67]), while it was negatively correlated with IMAT (−1.45, [−2.21, −0.70]) and SAT (−0.32, [−0.50, −0.13]). Gait speed was directly related to muscle area (0.01, [0.00, 0.02]) and inversely to IMAT (−0.04, [−0.07, −0.01]), while chair sit‐to‐stand time was associated with muscle area (0.98, [0.98, 0.99]), IMAT area (1.04, [1.01, 1.07]), SAT area (1.01, [1.01, 1.02]) and AHU/h^2^ for muscle (0.97, [0.95, 0.99]). MAT area showed a potential link to non‐trauma fractures post‐50 years (1.67, [0.98, 2.83]). Femoral neck BMD was associated with cortical bone (0.09, [0.08, 0.10]), MAT (−0.11, [−0.13, −0.10]), MAT adjusted for total bone marrow area (−0.06, [−0.07, −0.05]) and AHU/h^2^ for muscle (0.01, [0.00, 0.02]). Total spine BMD showed similar associations and with AHU for muscle (0.02, [0.00, 0.05]). Total lean mass was correlated with cortical bone (517.3, [148.26, 886.34]), trabecular bone (924, [262.55, 1585.45]), muscle (381.71, [291.47, 471.96]), IMAT (−1096.62, [−1410.34, −782.89]), SAT (−413.28, [−480.26, −346.29]), AHU (527.39, [159.12, 895.66]) and AHU/h^2^ (300.03, [49.23, 550.83]).

**Conclusion:**

Our deep learning–based technique offers a fast and accurate method for segmentation and quantification of musculoskeletal tissues in proximal hip, with potential clinical value.

## 1 Introduction

Bone and muscle loss can arise from diverse factors, encompassing age‐related conditions such as osteoporosis and sarcopenia, as well as chronic diseases like cancer, kidney disease and autoimmune disorders. These conditions profoundly influence the volume and composition of musculoskeletal tissues and predispose older adults to adverse events (i.e., falls and fractures), thereby substantially affecting individuals' overall quality of life and life expectancy [1] [S1].

Fragility fractures, especially hip fractures, are significant adverse outcomes of pathological changes in body composition [2]. Predicting and preventing these fractures is crucial. They are influenced by various factors, including tissue loss conditions. Muscle loss and weakness, which impair physical performance and balance, increase fall and fracture risks in those with weakened bones [S2]. Fatty infiltration in bones and muscles affects their mass and density, with studies showing a negative correlation between marrow adiposity and bone formation [S3]. Higher fat concentration in bones is common in osteoporosis [3], and muscle fat infiltration reduces muscle function [4]. Previous studies also highlight an interaction between fat deposition in bones and muscles [3]. Therefore, examining bone, muscle and fat, both individually and collectively, is vital for understanding the pathophysiology and clinical aspects of osteoporosis, sarcopenia and osteosarcopenia and for better prediction of adverse outcomes.

Computed tomography (CT) is a 3D imaging technique that accurately differentiates muscle, fat and bone. Moreover, CT scans are widely used to assess osteoporosis and sarcopenia and measure adiposity in bone and muscle [3] [S4]. Bone mineral density (BMD) independent muscle density measurements from CT scan have shown excellent predictive power for hip fractures and mortality [5] [S5]. CT parameters such as cross‐sectional area and average Hounsfield unit (AHU) from the abdomen and thigh areas are reliable tools for the assessment of the musculoskeletal system [6] [S6]. However, despite housing the body's largest muscles and the hip bone, the proximal hip region has been relatively understudied [7].

CT scan analysis, both quantitative and qualitative, is challenging due to complex image processing. Previous studies have used manual and semi‐automatic methods like thresholding, watershed and active contour for musculoskeletal tissue segmentation [8] [S7, S8], but these techniques are user‐dependent, time‐consuming and sensitive to noise. More automated methods, such as atlas‐based modelling, are effective for organ segmentation but require labour‐intensive and time‐consuming shape model construction [S9]. Hence, there is a need for more accurate and fully automated image analysis techniques.

Deep learning is a subset of machine learning, which itself is a branch of artificial intelligence (AI) that focuses on algorithms that enable computers to learn from and make predictions or decisions based on data [S10]. Deep learning distinguishes itself by using artificial neural networks with many layers, hence the term ‘deep’. Deep learning represents a pivotal advancement in musculoskeletal tissue segmentation because it can process and learn from complex, high‐dimensional data, surpassing traditional segmentation methods that often rely on manual delineation or rule‐based algorithms [9]. This relevance is underscored by deep learning's inherent capacity for feature extraction and pattern recognition, enabling the identification of complex textures and structures within medical images that are typically challenging for conventional methods [9]. Several studies have shown the successful application of deep learning methods for segmentation of muscle, bone and fat in key areas of the body such as mid‐thigh [S11], pelvis [S12] and abdomen [10] [S13, S14].

To the best of our knowledge, there is a notable research gap concerning developing a deep learning approach that comprehensively addresses the segmentation and quantification of musculoskeletal tissues, including marrow adipose tissue (MAT), in the proximal hip region. In this study, we tested a fully automated technique that addresses this gap by enabling the precise segmentation and quantification of bone, MAT, muscle, intermuscular adipose tissue (IMAT) and subcutaneous adipose tissue (SAT) in the proximal hip CT scan. We also aimed to investigate the clinical significance of quantitative measurements generated from our model by examining their correlation with clinically relevant indicators, including functional and strength variables such as grip strength, chair sit‐to‐stand time and walking speed, along with bone density in the femoral neck and spine, as well as whole body lean mass, all variables that, when affected, predispose older persons to falls and fractures.

## 2 Methods

### 2.1 Population and Data Source

Our study included 300 participants from the Osteoporotic Fractures in Men study (MrOS) (website: https://mrosonline.ucsf.edu), a multicentre project involving men aged 65 and older from three international cohorts with similar protocols. We randomly selected 200 participants from the Monongahela Valley near Pittsburgh Centre (PA) and 100 from the Hong Kong (HK) cohort. Eligibility required unassisted walking ability and no bilateral hip replacements [S18]. Selection of the number of participants in this study was influenced by the time available for manual segmentation of ground truth images for training of the deep learning model.

Baseline assessments included height measured on wall‐mounted stadiometers and weight measured on balance beam or digital scales, from which body mass index (BMI) was derived. Functional assessments were conducted, including the time taken to complete five chair sit‐to‐stands, walking speed over a 6‐m distance at the participant's usual pace and grip strength measured using JAMAR dynamometers (Sammons Preston Rolyan, Bolingbrook, IL, USA). Dual X‐ray absorptiometry (DXA) variables such as areal bone mineral density (BMD) and body composition were measured using Hologic QDR 4500 A or W (Hologic, Bedford, MA, USA) instruments [S19]. Additionally, information regarding previous fractures occurring after the age of 50 years and self‐reported falls during the 12 months prior to the baseline visit were collected through questionnaires.

### 2.2 Quantitative CT Images

Quantitative CT (QCT) scans of the hip were acquired using a Siemens Somatom Plus 4 scanner (Siemens, Munich, Germany) and GE Medical Systems/lightspeed 16 (Waukesha, WI, USA) in PA and HK studies, respectively. Measurements were taken in the pelvic region, covering from the femoral head to 3.5 cm below the lesser trochanter. The scan settings comprised 80 kVp, 280 mA, 3 mm slice thickness and a 512 × 512 matrix, utilizing spiral reconstruction mode. For calibration purposes, hydroxyapatite calibration standards with known concentrations (150, 75 and 0 mg/cm^3^) from Image Analysis Inc. (Columbia, KY, USA) were included in each scan along with the participant.

### 2.3 Manual Segmentation

Ground truth images for the 300 participants were generated through interactive pixel segmentation and thresholding techniques using Slice‐O‐Matic software (TomoVision, Montreal, CA). The slices with the thickest left femoral neck were selected to establish the reference slice. For volumetric analysis, two slices above and below the reference slice were chosen (five slices in total). Within each slice, seven tissues were segmented, namely, cortical bone, trabecular bone, haematopoietic bone marrow (HBM), MAT, muscle, IMAT and SAT. Two trained medical students, T.D. and J.B., performed the manual segmentation. To assess inter‐rater reliability, J.B. repeated the segmentation of 20 images initially segmented by T.D.

The Hounsfield unit (HU) threshold values for cortical bone were derived by measuring the thresholds at the femoral shaft, which predominantly consists of cortical bone (> 300). Trabecular bone thresholds were calculated by subtracting the cortical bone threshold from the existing thresholds for low‐density (cancellous) bone (150–300) [S20]. MAT thresholds were determined by measuring the thresholds for the spinal cord, which shares similar characteristics with MAT (fat surrounded by bone) (< 50). The HBM threshold was defined as the difference between trabecular bone and MAT (50–150). Threshold values for muscle (−30 to 500) and fat (employed for both IMAT and SAT [< −30]) were obtained from previous studies [S15].

### 2.4 Automatic Segmentation

#### 2.4.1 Deep Learning Model

The U‐Net architecture [11] is a widely used deep learning model for segmenting medical images. It consists of a contracting path (encoder) and an expansive path (decoder) with skip connections between them. The contracting path captures features from the input image, while the expansive path up‐samples the features to generate a probability mask indicating tissue segmentation. The skip connections notably preserve fine details, which are crucial for medical image segmentation. In this study, Dense U‐Net, which is a variant of U‐Net architecture, was selected. In Dense U‐Net, traditional U‐Net blocks are replaced with dense blocks, enhancing the model's ability to distinguish closely located and overlapping tissues [12]. A schematic representation of the architecture used in this study is included in Figures S1 and S2.

#### 2.4.2 Training Procedure

The models in this study were trained using mini‐batch stochastic gradient descent with Adam as the optimizer. A batch size of 2 was chosen, and a learning rate of 0.0001 was applied. The training process was conducted for 200 epochs. Hyperparameter values were tuned empirically to achieve optimal performance. For this study, categorical cross‐entropy loss function was selected [S16]. All experiments were conducted using Python 3.7.13, TensorFlow 2.8.2 and Keras 2.8.0 open‐source software.

#### 2.4.3 Model Performance Evaluation

Model performance was evaluated using the Dice similarity coefficient (DSC), average symmetric surface distance (ASSD), sensitivity and specificity. The DSC quantifies the spatial overlap between the ground truth labels (A)—tissues that were manually annotated by a trained user—and the predicted labels (B), which represent the model‐generated tissue predictions. It is calculated as (A,B) = 2(A∩B)/(A + B), where ∩ represents the intersection [S17]. The DSC ranges from 0 to 1, with a value of 1 indicating perfect agreement between the labels. In this study, DSC was calculated for each tissue type individually and is presented as a percentage. Moreover, the ASSD is used to measure the average distance between voxels on the boundary of the predicted labels and the corresponding voxels on the boundary of the ground truth labels [S21]. A lower ASSD indicates a closer match between the prediction and ground truth labels. The ASSD was calculated for each tissue separately and measured in millimetres (mm).

Sensitivity, or the true positive rate, measures the proportion of actual positives (e.g., correctly identified tissue segments) that are correctly identified by the model. Specifically, for each tissue class, sensitivity was calculated as the number of true positive identifications divided by the sum of true positives and false negatives, indicating the model's ability to correctly identify each tissue type. On the other hand, specificity, or the true negative rate, assesses the model's ability to correctly identify negatives (e.g., correctly indicating the absence of a specific tissue type). For each class, specificity was calculated as the number of true negatives divided by the sum of true negatives and false positives.

In this study, k‐fold cross‐validation was also employed to evaluate the models. This technique involves dividing the dataset into k subsets or folds. The models were trained using *k* − 1 subsets, and the remaining subset was used for evaluation. This process was repeated k times, ensuring each subset served as the evaluation set. Lastly, the results from each fold were averaged to obtain a comprehensive assessment of model performance. Our study utilized *k* = 10.

To assess the model's speed, we measured the time taken for manually segmenting one slice for 100 patients. This time was then compared with the time required for the deep learning model's automated segmentation of the same set of slices. We used a Windows 10–powered personal computer with an Intel Core I7 CPU processor, 16 GB RAM and a 64‐bit operating system to generate segmentation masks using the deep learning model.

### 2.5 Robustness Experiment

To evaluate the effect of dataset size on model generalizability and robustness, two sets of models were trained. One set of models was trained on the PA dataset, while the other was trained on a combined dataset of PA and HK (PA + HK). From the PA‐trained models, the best‐performing model was selected and tested on the HK dataset (considered new, unseen data) and on a subset of unseen data from the PA dataset (10% of the total, held out during training as a test set). Similarly, the best‐performing model from the PA + HK‐trained models was selected, and its test results were compared to those from the PA‐trained model.

### 2.6 Statistical Methods

Participant characteristics are presented as medians (interquartile range, IQR) and counts (percentages). Pearson's correlation was used to assess the association between quantitative measures from deep learning models (tissue area and volume). Linear regression was used to evaluate the associations between quantitative measures and clinical characteristics (grip strength, gait speed, chair sit‐to‐stand time and DXA measurements), while logistic regression was used for history of falls and fractures. Chair sit‐to‐stand time was log‐transformed to normalize data distribution. Models were also adjusted for pre‐specified confounders—age, height and weight. Results are presented as coefficients or odds ratios (OR) with 95% confidence intervals. All analysis was performed using Stata 17 (StataCorp LLC, College Station, TX).

## 3 Results

### 3.1 Segmentation Results

**FIGURE 1:**
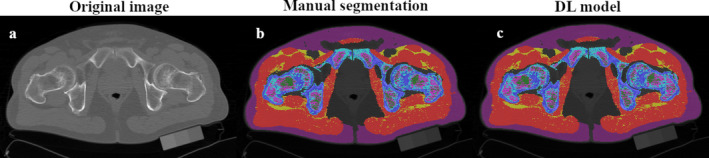
The figure represents an example of accurate segmentation performed by the deep learning model compared to manual segmentation.

Two sets of models were trained on the PA dataset (200 slices) and PA + HK dataset (300 slices) and tested on unseen data from the same datasets. DSC, ASSD, sensitivity and specificity were calculated for each tissue type studied. The results are presented in Table 1 as average and standard deviation of 10‐fold cross validation technique. Both models demonstrated high accuracy across test datasets, with an average DSC of 90.9% for models trained on the PA dataset and 90% for those trained on the combined PA + HK dataset (Figure 1).

**TABLE 1:** Dice similarity coefficient (DSC), average symmetric surface distance (ASSD), sensitivity and specificity of the deep learning models when trained on PA and PA + HK datasets.

Training dataset	Testing dataset	Tissues	DSC (%)	ASSD (mm)	Sensitivity (%)	Specificity (%)
PA (*n* = 180)	PA (*n* = 20)	Cortical bone	96.3 ± 1.82	0.19 ± 0.11	96 ± 2.23	99.93 ± 0.11
		Trabecular bone	88.37 ± 4.64	0.22 ± 0.1	88.95 ± 5.5	99.82 ± 0.05
		HBM	86.93 ± 3.93	0.35 ± 0.33	88.41 ± 5.37	99.89 ± 0.02
		MAT	88.85 ± 3.01	0.54 ± 0.47	90.46 ± 6.8	99.96 ± 0.01
		Muscle	96.02 ± 0.72	0.46 ± 0.1	96.15 ± 0.88	99.6 ± 0.1
		IMAT	84.36 ± 2.45	0.72 ± 0.24	85.1 ± 4.39	99.7 ± 0.6
		SAT	95.47 ± 1.55	0.74 ± 0.24	96.54 ± 1.77	99.64 ± 0.11
PA + HK (*n* = 270)	PA + HK (*n* = 30)	Cortical bone	96.86 ± 0.78	0.15 ± 0.04	95.81 ± 1.32	99.96 ± 0.01
		Trabecular bone	88.99 ± 2.4	0.21 ± 0.06	89.76 ± 2.37	99.84 ± 0.02
		HBM	86.51 ± 2.73	0.28 ± 0.09	88.54 ± 3.23	99.88 ± 0.02
		MAT	88.76 ± 2.31	0.42 ± 0.13	91.13 ± 4.67	99.95 ± 0.01
		Muscle	93.46 ± 3.52	0.83 ± 0.55	93.27 ± 3.5	99.68 ± 0.03
		IMAT	81 ± 2.6	1.05 ± 0.43	82.19 ± 3.44	99.73 ± 0.1
		SAT	94.23 ± 1.97	0.05 ± 0.5	96.35 ± 0.88	99.41 ± 0.4

*Note:* The results are presented as the average ± standard deviation of 10‐fold cross validation.

Abbreviations: HBM: hematopoietic bone marrow; HK: Hong Kong dataset; IMAT: intermuscular adipose tissue; MAT: marrow adipose tissue; PA: Monongahela Valley near Pittsburgh Centre dataset; SAT: subcutaneous adipose tissue.

To demonstrate the effect of a larger dataset on the generalizability of the model, the results from testing the best‐performing PA model on both the unseen PA dataset and unseen HK dataset, as well as the results from testing the best‐performing PA + HK model on test set of unseen PA + HK dataset has been presented in Table 2. A decrease in the performance of the PA model was observed when tested on the HK dataset from an average DSC of 93.45%–85.13% over all tissues. On the other hand, an increase was observed in the accuracy of the models when the models were trained on PA + HK datasets compared to models trained only on PA dataset (average DSC of 92.48%).

**TABLE 2:** Dice similarity coefficient (DSC), average symmetric surface distance (ASSD), sensitivity and specificity of the deep learning models when trained on PA dataset and tested on PA and HK dataset and when trained and tested on PA + HK dataset.

Training dataset	Testing dataset	Tissues	DSC (%)	ASSD (mm)	Sensitivity (%)	Specificity (%)
PA (*n* = 180)	PA (*n* = 20)	Cortical bone	97.97	0.08	98.02	99.96
		Trabecular bone	93	0.12	93.44	99.88
		HBM	91.82	0.16	92.65	99.92
		MAT	92.76	0.26	92.56	99.97
		Muscle	96.93	0.37	96.35	99.75
		IMAT	85.65	0.64	86.17	99.82
		SAT	96.06	0.55	96.57	99.76
	HK (*n* = 100)	Cortical bone	95.91	0.43	95.54	99.92
		Trabecular bone	85.25	0.59	89.28	99.69
		HBM	82.16	0.48	84.68	99.87
		MAT	82.18	0.99	84.07	99.96
		Muscle	92.69	1.24	93.43	99.32
		IMAT	69.46	2.13	71.31	99.68
		SAT	88.28	2.14	89.21	99.38
PA + HK (*n* = 270)	PA + HK (*n* = 30)	Cortical bone	97.77	0.09	97.18	99.96
		Trabecular bone	91.86	0.14	92.08	99.87
		HBM	90.40	0.16	92.46	99.89
		MAT	92.04	0.20	93.98	99.96
		Muscle	96.45	0.42	96.64	99.65
		IMAT	83.39	0.70	82.80	99.83
		SAT	95.49	0.69	96.97	99.68

*Note:* The results are presented for the best performing model from 10‐fold cross validation.

Abbreviations: HBM: hematopoietic bone marrow; HK: Hong Kong dataset; IMAT: intermuscular adipose tissue; MAT: marrow adipose tissue; PA: Monongahela Valley near Pittsburgh Centre dataset; SAT: subcutaneous adipose tissue.

The average time required to segment one slice was measured for both manual segmentation and automatic segmentation using the PA + HK model across 100 slices. The average time for manual segmentation was 15.5 min compared to 6.5 s for automated segmentation. Inter‐rater reliability was excellent, with a DSC above 98% across all tissues for 20 patients.

### 3.2 Clinical Results

#### 3.2.1 Demographics, Medical History and Clinical Parameters

The HK centre participants were relatively younger, with a median age of 70 years compared to 73 years at the PA centre. Similarly, the HK group exhibited a lower BMI (23.3 kg/m^2^) than the PA group (25.3 kg/m^2^). The prevalence of health conditions also varied, with lower rates of diabetes, osteoporosis, stroke, arthritis, cancer and osteoarthritis in the HK cohort, but higher rates of hypertension and chronic obstructive pulmonary disease. Smoking rates were notably higher in HK, while fracture occurrences were lower. Falls were more common in the HK group. HK participants had lower grip strength and walking speed and higher chair sit‐to‐stand time. BMD was lower in the femoral neck and spine for HK individuals, and total lean mass was also lower among them compared to the PA participants. Detailed comparisons are available in Table 3.

**TABLE 3:** Demographics, medical history and clinical parameters for HK and PA datasets.

		HK dataset	PA dataset	Total	*p*
	Number of participants	100	200	300	
General characteristics	Age at enrolment (years)^a^	70.00 (67.00, 73.50)	73.00 (69.00, 77.00)	72.00 (68.00, 75.00)	< 0.001
	Race	100% Asian	87% Caucasian		
	Height (cm)^a^	163.00 (158.90, 166.88)	173.75 (168.90, 178.50)	170.15 (163.90, 176.30)	< 0.001
	Weight (kg)^a^	62.15 (56.65, 66.50)	75.90 (69.35, 83.45)	71.20 (63.90, 80.45)	< 0.001
	BMI (kg/m^2^)^a^	23.32 (21.41, 25.50)	25.33 (23.53, 26.99)	24.72 (22.79, 26.56)	< 0.001
History of chronic conditions	Diabetes	16 (16.0%)	12 (6.0%)	28 (9.3%)	0.010
	Osteoporosis	5 (5.0%)	7 (3.5%)	12 (4.0%)	0.54
	Stroke	6 (6.0%)	8 (4.0%)	14 (4.7%)	0.56
	Hypertension	49 (49.0%)	62 (31.0%)	111 (37.0%)	0.003
	COPD	15 (15.0%)	18 (9.0%)	33 (11.0%)	0.12
	Arthritis or gout	21 (21.0%)	88 (44.0%)	109 (36.3%)	< 0.001
	Cancer	4 (4.0%)	54 (27.0%)	58 (19.3%)	< 0.001
	Osteoarthritis	4 (19%)	44 (50%)	48 (69%)	0.222
Cigarette smoking history	Smoked 100 cigarettes in life	69 (69.0%)	116 (58.0%)	185 (61.7%)	0.078
Cigarette smoking status	Never smoked	31 (31.0%)	84 (42.0%)	115 (38.3%)	
	Ex‐smoker	57 (57.0%)	114 (57.0%)	171 (57.0%)	
	Current smoker	12 (12.0%)	2 (1.0%)	14 (4.7%)	
Falls and fracture history	Ever broke or fractured a bone	12 (12.0%)	111 (55.5%)	123 (41.0%)	< 0.001
	Non‐trauma fracture after age of 50	5 (5.0%)	37 (18.5%)	42 (14.0%)	0.001
	Fallen in the past 12 months	11 (89.0%)	49 (24.5%)	60 (20.0%)	0.006
Functional characteristic	Avg of right/left grip strength (kg)^a^	31.25 (27.25, 35.75)	40.00 (35.00, 45.25)	36.75 (31.00, 42.00)	< 0.001
	Seconds to complete 5 chair stands^a^	12.28 (10.78, 14.19)	9.97 (8.69, 12.03)	10.86 (9.16, 12.85)	< 0.001
	Walk speed using best time (m/s)^a^	1.06 (0.93, 1.21)	1.33 (1.19, 1.46)	1.25 (1.08, 1.40)	< 0.001
DXA measurements	Femoral neck BMD (g/cm^2^)^a^	0.69 (0.62, 0.75)	0.76 (0.69, 0.85)	0.74 (0.65, 0.81)	< 0.001
	Total spine BMD (g/cm^2^)^a^	0.94 (0.79, 1.06)	1.04 (0.90, 1.17)	1.01 (0.88, 1.12)	< 0.001
	Total lean mass (g)^a^	44 042.28 (41 459.47, 47 006.43)	54 929.66 (50 395.76, 58 468.63)	51 208.01 (45 481.62, 56 850.92)	< 0.001

Abbreviations: BMD: bone mineral density; BMI: body mass index; COPD: chronic obstructive pulmonary disease; DXA: dual X‐ray absorptiometry; HK: Hong Kong dataset; PA: Monongahela Valley near Pittsburgh Centre dataset. *p*‐values significant at *p* < 0.05.

^a^ Values shown as median and the interquartile range: median (first quartile, third quartile).

#### 3.2.2 Model's Quantitative Measurements

Quantitative measurements from the deep learning model are shown in Table 4. There was a weak correlation among area measures, with strongest correlation observed between total bone marrow and MAT (*r* = 0.754), cortical bone and total bone marrow (*r* = −0.584) and cortical bone and MAT (*r* = −0.588) (Figure S3). A strong correlation was observed between area and volume measurements across both centres, so only area was used in subsequent analysis (Figure S4).

**TABLE 4:** Association between quantitative measurements generated by deep learning model from PA and HK datasets.

	Studied tissues	HK dataset (*n* = 100)	PA dataset (*n* = 200)	Total	*p*
Volume for 5 slices (cm^3^)	Cortical bone	27.32 (24.39, 31.22)	38.43 (33.63, 43.00)	0.71 (0.64, 0.77)	< 0.001
	Trabecular bone	18.03 (16.66, 20.24)	25.65 (23.47, 28.64)	0.29 (0.23, 0.36)	< 0.001
	HBM	9.72 (8.02, 11.52)	13.82 (10.58, 16.17)	34.70 (28.95, 40.93)	< 0.001
	MAT	3.90 (2.35, 5.89)	5.85 (3.68, 9.13)	23.67 (19.63, 26.76)	< 0.001
	Muscle	108.07 (94.33, 116.86)	164.99 (153.40, 177.91)	11.94 (9.13, 15.11)	< 0.001
	IMAT	15.09 (11.37, 20.55)	24.75 (18.67, 32.87)	5.24 (3.13, 7.70)	< 0.001
	SAT	87.72 (74.81, 107.65)	116.76 (95.33, 145.26)	153.40 (116.86, 172.12)	< 0.001
Area (cm^2^)	Cortical bone	45.81 (40.13, 52.84)	48.50 (41.84, 55.50)	21.23 (15.42, 28.89)	0.050
	Trabecular bone	30.06 (27.48, 33.84)	32.76 (29.63, 36.86)	105.45 (84.20, 136.79)	< 0.001
	HBM	15.84 (12.60, 19.49)	17.97 (13.59, 21.65)	47.85 (41.23, 54.81)	0.004
	MAT	6.42 (3.68, 9.59)	7.88 (4.32, 12.12)	32.10 (28.90, 36.09)	0.056
	Muscle	172.54 (152.32, 187.64)	206.24 (191.15, 224.20)	17.25 (13.19, 20.78)	< 0.001
	IMAT	23.93 (17.86, 32.99)	31.05 (23.62, 41.02)	7.25 (4.14, 11.46)	< 0.001
	SAT	140.05 (116.00, 172.78)	144.63 (118.12, 179.69)	196.36 (175.64, 216.88)	0.50

*Note:* The results are presented as median and interquartile range: median (first quartile, third quartile). *p*‐value significant at *p* < 0.05.

Abbreviations: AHU: average Hounsfield unit; HBM: haematopoietic bone marrow; HK: Hong Kong dataset; IMAT: intermuscular adipose tissue; MAT: marrow adipose tissue; PA: Monongahela Valley near Pittsburgh Centre dataset; SAT: subcutaneous adipose tissue; TBM: total bone marrow.

#### 3.2.3 Association Between Quantitative Measurements and Function and Strength Characteristics

In this section, deep learning–generated quantitative measurements have been treated as exposure and function and strength variables including grip strength, gait speed and five chair sit‐to‐stand time as well as likelihood of non‐trauma fractures and the history of falls (presented as odds ratio) were considered outcome variables.

Higher grip strength was significantly associated with higher cortical bone area (coefficient and 95% confidence interval of 0.95, [0.10, 1.80]), higher muscle area (0.41, [0.19, 0.64]), lower IMAT (−1.45, [−2.21, −0.70]), lower SAT (−0.32, [−0.50, −0.13]) and higher average HU adjusted for height squared (AHU/h^2^) for muscle (1.1, [0.53, 1.67]) (Table 5).

**TABLE 5:** Association between quantitative measurements generated by deep learning model and strength and functional variables.

	Grip strength (kg)			Gait speed (m/s)			Five chair sit‐to‐stand time (s)		
Studied tissues	Coefficient	95% CI	*p*	Coefficient	95% CI	*p*	Exp coefficient	95% CI	*p*
Cortical bone	0.95	[0.10, 1.80]	0.029	−0.01	[−0.04, 0.02]	0.595	0.99	[0.95, 1.02]	0.388
Trabecular bone	−0.19	[−1.73, 1.35]	0.812	−0.03	[−0.08, 0.02]	0.282	0.99	[0.94, 1.06]	0.866
HBM	−0.55	[−1.99, 0.89]	0.453	0	[−0.05, 0.05]	0.858	1.03	[0.98, 1.09]	0.264
MAT	−0.48	[−1.81, 0.86]	0.483	0	[−0.05, 0.04]	0.849	1.05	[1.00, 1.11]	0.069
Muscle	0.41	[0.19, 0.64]	< 0.001	0.01	[0.00, 0.02]	0.002	0.98	[0.98, 0.99]	0.001
IMAT	−1.45	[−2.21, −0.70]	< 0.001	−0.04	[−0.07, −0.01]	0.003	1.04	[1.01, 1.07]	0.009
SAT	−0.32	[−0.50, −0.13]	0.001	−0.01	[−0.01, −0.00]	0.014	1.01	[1.01, 1.02]	0.001
HBM/TBM	0.34	[−0.43, 1.11]	0.388	0	[−0.02, 0.03]	0.761	0.97	[0.94, 1.00]	0.041
MAT/TBM	−0.34	[−1.11, 0.43]	0.388	0	[−0.03, 0.02]	0.761	1.03	[1.00, 1.06]	0.041
AHU muscle	0.8	[−0.05, 1.65]	0.066	−0.01	[−0.04, 0.02]	0.355	0.98	[0.94, 1.01]	0.148
AHU muscle/h^2^	1.1	[0.53, 1.67]	< 0.001	0.01	[−0.00, 0.03]	0.099	0.97	[0.95, 0.99]	0.001

*Note:* Chair sit‐to‐stand time was log transformed; Exp coefficients represents fold difference in chair sit‐to‐stand time. The results are presented as coefficient and 95% confidence interval. *p*‐value significant at *p* < 0.05. The results are presented for the best performing model trained on PA + HK datasets.

Abbreviations: AHU: average Hounsfield unit; CI: confidence interval; HBM: haematopoietic bone marrow; HK: Hong Kong dataset; IMAT: intermuscular adipose tissue; MAT: marrow adipose tissue; PA: Monongahela Valley near Pittsburgh Centre dataset; SAT: subcutaneous adipose tissue; TBM: total bone marrow.

Higher gait speed was associated with higher muscle area (0.01, [0.00, 0.02]) and lower IMAT (−0.04, [−0.07, −0.01]). Moreover, higher chair sit‐to‐stand time was associated with lower muscle area (0.98, [0.98, 0.99]), higher IMAT area (1.04, [1.01, 1.07]), higher SAT area (1.01, [1.01, 1.02]) and lower AHU/h^2^ for muscle (0.97, [0.95, 0.99]) (Table 5).

Only a higher MAT area showed some association with a higher likelihood of non‐trauma fractures after the age of 50 years (1.67, [0.98, 2.83] and *p*‐value = 0.057). No variables were associated with the history of falls.

#### 3.2.4 Association Between Quantitative Measurements and DXA Measurements

In this section, deep learning–generated quantitative measurements have been treated as exposure, and DXA measurements including BMD of femoral, BMD of total spine and total lean mass have been treated as outcome variables.

Higher femoral neck BMD was significantly associated with a higher cortical bone area (0.09, [0.08, 0.10]), lower MAT area (−0.11, [−0.13, −0.10]), lower MAT area adjusted for total bone marrow area (−0.06, [−0.07, −0.05]) and higher AHU/h^2^ for muscle (0.01, [0.00, 0.02]). Similar to femoral neck BMD, higher total spine BMD was significantly associated with higher cortical bone area (0.12, [0.10, 0.14]), lower MAT area (−0.14, [−0.17, −0.11]), lower MAT area adjusted for total bone marrow area (−0.08, [−0.10, −0.06]) and higher AHU for muscle (0.02, [0.00, 0.05]) (Table 6).

**TABLE 6:** Association between quantitative measurements generated by deep learning model and DXA variables.

	Corrected femoral neck BMD (g/cm^2^)			Total spine BMD (g/cm^2^)			Total lean mass (g)		
	Coefficient	95% CI	*p*	Coefficient	95% CI	*p*	Coefficient	95% CI	*p*
Cortical bone	0.09	[0.08, 0.10]	< 0.001	0.12	[0.10, 0.14]	< 0.001	517.3	[148.26, 886.34]	0.006
Trabecular bone	−0.01	[−0.04, 0.01]	0.229	−0.02	[−0.06, 0.02]	0.257	924	[262.55, 1585.45]	0.006
HBM	−0.12	[−0.14, −0.10]	< 0.001	−0.14	[−0.17, −0.11]	< 0.001	253.07	[−373.51, 879.64]	0.427
MAT	‐0.11	[−0.13, −0.10]	< 0.001	−0.14	[−0.17, −0.11]	< 0.001	−181.27	[−764.03, 401.48]	0.541
Muscle	0	[−0.00, 0.01]	0.131	0	[−0.00, 0.01]	0.428	381.71	[291.47, 471.96]	< 0.001
IMAT	−0.01	[−0.02, 0.01]	0.338	−0.01	[−0.03, 0.00]	0.14	−1096.62	[−1410.34, −782.89]	< 0.001
SAT	0	[−0.01, 0.00]	0.091	0	[−0.01, 0.00]	0.34	−413.28	[−480.26, −346.29]	< 0.001
HBM/BM	0.06	[0.05, 0.07]	< 0.001	0.08	[0.06, 0.10]	< 0.001	245.48	[−92.42, 583.37]	0.154
MAT/BM	−0.06	[−0.07, −0.05]	< 0.001	−0.08	[−0.10, −0.06]	< 0.001	−245.48	[−583.37, 92.42]	0.154
AHU muscle	0.01	[−0.00, 0.02]	0.119	0.02	[0.00, 0.05]	0.033	527.39	[159.12, 895.66]	0.005
AHU muscle/h^2^	0.01	[0.00, 0.02]	0.021	0.01	[−0.00, 0.03]	0.062	300.03	[49.23, 550.83]	0.019

*Note:* The results are presented as coefficient and 95% confidence interval. The results are presented for the best performing model trained on PA + HK datasets. *p*‐value significant at *p* < 0.05.

Abbreviations: AHU: average Hounsfield unit; CI: confidence interval; DXA: dual X‐ray absorptiometry; HBM: haematopoietic bone marrow; HK: Hong Kong dataset; IMAT: intermuscular adipose tissue; MAT: marrow adipose tissue; PA: Monongahela Valley near Pittsburgh Centre dataset; SAT: subcutaneous adipose tissue; TBM: total bone marrow.

Higher total lean mass was significantly associated with a higher cortical bone area (517.3, [148.26, 886.34]), higher trabecular bone area (924, [262.55, 1585.45]), higher muscle area (381.71, [291.47, 471.96]), lower IMAT area (−1096.62, [−1410.34, −782.89]), lower SAT area (−413.28, [−480.26, −346.29]), higher AHU for muscle (527.39, [159.12, 895.66]) and higher AHU/h^2^ for muscle (300.03, [49.23, 550.83]) (Table 6).

## 4 Discussion

This study demonstrates a deep learning–based approach for precise, automated segmentation and quantification of musculoskeletal tissues in CT scans of the proximal hip region. We also assessed our approach's robustness by successfully applying it to data from two distinct international cohorts. Moreover, our investigation extends to the clinical utility of our model by establishing associations between the quantitative outputs from the deep learning model and clinically relevant variables, including assessments of muscle function and strength, as well as measurements obtained via DXA. Our study is novel in demonstrating the feasibility of quantifying key components, such as muscle and fat adiposity, within the proximal hip area—an important region in understanding sarcopenia and osteoporosis.

CT is regarded as a standard technique for the assessment of body composition as well as tissue loss conditions such as osteoporosis, sarcopenia, osteosarcopenia, malnutrition, COPD, cachexia, diabetes and renal failure [13]. Although previously the use of CT scans has been considered suboptimal due to radiation exposure and labour‐intensive image segmentation, new CT technologies have reduced the radiation exposure by utilizing various reconstruction and deep learning–based denoising techniques [S22, S23]. Moreover, CT scans are cheaper, faster and more comfortable for patients than MRI [14].

Deep learning techniques offer a fast, accurate tool for segmenting musculoskeletal tissues in CT scans. Several studies have focused on segmentation of muscle, bone and fat in the abdomen [15, 16, 17, 18] [S24, S25, S26] and thigh [19] [S27, S28] area, but the number of studies investigating the hip area is limited. Nishiyama et al. utilized a deep generative model to effectively segment the gluteus medius muscle in CT scans [20]. Hiasa et al. and Iwasa et al. successfully segmented individual muscles in hip and thigh CT scans using Bayesian U‐Net models [21] [S29]. Other studies achieved muscle IMAT segmentation in hip CT scans [7] and muscle and SAT segmentation in both abdominal and hip areas [22]. To our knowledge, no other study has presented a technique for evaluating body composition and bone (including bone marrow adiposity) in the proximal hip region or correlated these measurements with clinically relevant variables.

The proximal hip, a key anatomical area, contains vital musculoskeletal structures. It houses the femur, the largest bone, crucial for weight‐bearing, movement and daily activities [S30]. Assessing the proximal femur is essential due to its role in bone density evaluation and vulnerability to osteoporotic fractures, a major health concern [S31]. This region also includes large muscles like the gluteals, hip flexors, abductors and rotators, crucial for stability, locomotion and functional mobility [S32]. Interestingly, muscle density in the proximal hip better predicts hip fractures than traditional BMD measures [5] [S32]. Notably, the size and density of muscles in this region have been linked to post‐hip fracture mortality rates, underscoring their clinical significance [23]. Additionally, high IMAT volume in this area is associated with impaired balance and gait in older adults, highlighting the proximal hip's comprehensive role in musculoskeletal health [24].

**FIGURE 2:**
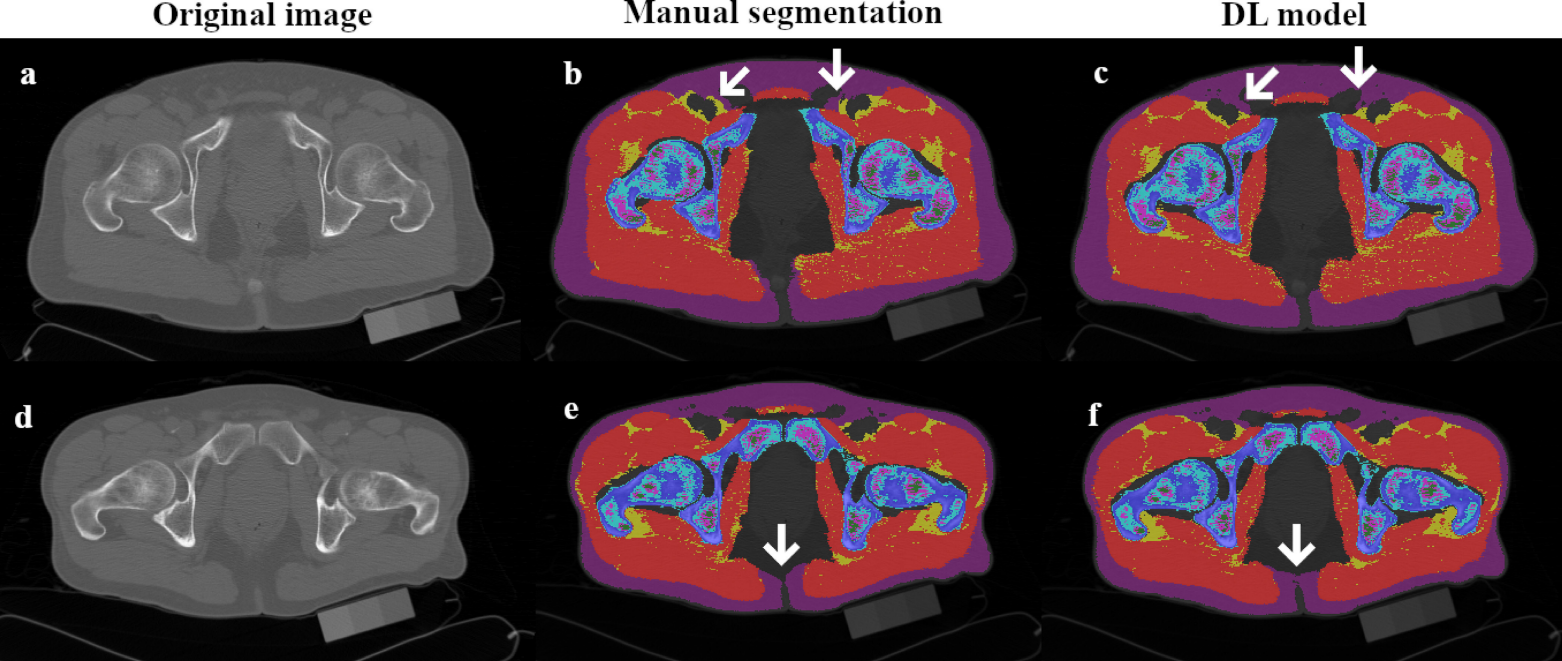
Examples of the model's high‐accuracy segmentation with minimal errors.

Our automatic segmentation method achieved accuracy comparable to other methods for musculoskeletal tissue assessment. For comparison, Barnard et al. achieved a DSC of 94% and 86% for psoas muscle segmentation at the L3 level [25], while Weston et al. reported DSCs of 93%, 88%, and 95% for SAT, muscle and bone, also at the L3 level [17]. Hemke et al. found DSCs of 97%, 95%, 91% and 92% for SAT, muscle, IMAT and bone in the pelvic area [26]. Notably, our model was trained on fewer images than some of these studies. Also, many studies did not use k‐fold cross‐validation, and their results reflect their best models. Our approach uniquely differentiates between cortical bone, trabecular bone, HBM and MAT, unlike others that group these as ‘bone’. Lower accuracy in tissues like IMAT and MAT is expected due to their smaller, more dispersed nature. Minor pixel changes can significantly affect DSC, as seen in previous research [17]. Figure 2 shows examples of our model's high‐accuracy segmentation with minimal errors.

To evaluate our technique's robustness, we tested the best model from the PA dataset on both PA and HK datasets. Despite an 8.32% drop in average DSC across all tissues, the model accurately segmented new data from a different imaging system (Table 2), demonstrating the method's transferability. Additionally, training the model with combined PA and HK data improved segmentation accuracy, with a 7.35% increase in average DSC across tissues (Table 2). This inclusion of diverse data sources enhanced the model's performance and generalizability.

Ageing increases fat accumulation in non‐adipose tissues like bone marrow, skeletal muscle and liver [27]. Factors like extended bed rest or space travel can worsen bone fat infiltration [28] [S33], and prolonged inactivity may lead to increased muscle fat and reduced strength, raising the risk of balance issues and falls [29] [S34]. This risk is heightened with decreased physical activity in older adults. Fat in lower limb muscles is linked to a higher fracture risk in older adults [30]. Additionally, fat infiltration in muscles and bones correlates with the severity of osteoporosis and sarcopenia, underscoring the need to study the interplay between fat deposition, strength and performance [3].

In muscle fatty infiltration, two main pathways are identified: IMAT and intramyocellular lipid (IMCL). IMAT involves fat accumulation within muscle bundles, while IMCL refers to fat within myofibers [31]. Our study effectively segmented IMAT, but direct IMCL segmentation using QCT scans is challenging due to technical limitations. Instead, the AHU can act as a surrogate for IMCL, as fat's lower HU value reduces muscle AHU. We found a significant link between lower AHU and AHU/h^2^ in muscle (higher IMCL), and reduced BMD in the femoral neck and spine, consistent with previous research on fatty muscle infiltration's impact on BMD [32]. Yin et al. reported a negative association between fat in the gluteus maximus and thigh muscles and proximal femur BMD [33], and Li et al. observed a similar correlation between IMAT, IMCL in the proximal femur and femur BMD [34].

Much like the presence of fat infiltration in muscle, our study also revealed a strong association between increased MAT, MAT adjusted for total bone marrow and lower BMD in both the femoral neck and spine. This finding aligns with prior research demonstrating a negative correlation between MAT and bone volume and density. Shen et al. reported an inverse relationship between MAT and BMD in older women [35]. Furthermore, Bani Hassan et al. extended this observation to older men, confirming a negative correlation between MAT volume and BMD [36].

Our study found a significant link between higher IMAT and lower AHU and AHU/h^2^ in muscle, correlating with decreased lean mass, lower grip strength and gait speed and longer chair sit‐to‐stand times. This suggests that fat accumulation in muscle adversely affects muscle mass, strength and function, likely due to local inflammatory and lipotoxic milieu [32]. These findings are consistent with previous studies: Beavers et al. and Murphy et al. noted a negative impact of IMAT on gait speed in older adults [37] [S35]. Khoja et al. observed that higher IMAT and lower AHU were linked to slower gait and longer chair sit‐to‐stand times in rheumatoid arthritis patients [38]. Perkisas et al. and Lim et al. found negative correlations between IMAT and grip strength and gait speed, respectively [39] [S36].

The effect of fat accumulation on musculoskeletal health has been previously studied. The excessive adipose tissue in bone and muscle is associated with the release of pro‐inflammatory molecules, which can impair bone and muscle metabolism [32]. Furthermore, insulin resistance itself contributes to these inflammatory responses, creating a feedback loop that further exacerbates metabolic dysfunction and potentially impacts musculoskeletal health [32] [S37].

We found that larger muscle areas were associated with increased lean mass, grip strength and better chair sit‐to‐stand performance, suggesting that muscle area measurements in the proximal hip are effective proxies for overall muscle mass, strength and function. This is in line with previous studies in different body regions. Morrell et al. showed that psoas muscle area could indicate whole body lean mass in haemodialysis patients [40], and Byun et al. found correlations between psoas muscle area, appendicular lean mass and grip strength, highlighting its usefulness in assessing conditions like sarcopenia [S38].

In our investigation, elevated MAT levels were associated with the occurrence of non‐trauma fractures in individuals aged 50 and above. This finding aligns well with the correlation between higher MAT and lower BMD in the femoral neck and spine. However, our analysis did not reveal any significant associations between other quantitative metrics and the incidence of falls or fractures. It is essential to acknowledge that this observed outcome might be attributed to the relatively constrained participant pool, given that the study population exhibited incidences of falls and fractures at rates of 20% and 14%, respectively.

Our study has several strengths. First, despite limited available data, we have successfully developed an accurate and efficient segmentation and quantification method for evaluating musculoskeletal tissues within the proximal hip region. Second, by examining data from two distinct cohorts, we have assessed the robustness and adaptability of our technique. Additionally, our study delves into the clinical significance of quantitatively assessing musculoskeletal tissues in the proximal hip by correlating the results generated from deep learning model with clinically relevant variables, indicating its potential impact on prognosis and diagnosis of conditions such as osteoporosis and sarcopenia in an opportunistic manner.

Our study does have certain limitations. First, our dataset includes only men; future studies should include women to provide a more comprehensive perspective. Furthermore, our study is based solely on cross‐sectional data. Longitudinal data would provide valuable insights into how musculoskeletal tissues change over time and could further enhance our understanding of these complex relationships. Moreover, while we have significantly improved model performance by incorporating data from a second dataset, we must acknowledge that we did not have access to a third to further assess the robustness of the technique. Expanding our analysis to include such additional datasets in the future would strengthen the generalizability and reliability of our segmentation model across various populations and scanning conditions. This study's population consists mostly of White participants from PA and Chinese participants residing in Hong Kong (HK); thus, generalizability to other races and ethnic groups remains uncertain. Further, our model included images from just two scanners, it is not clear how it would perform with images acquired from other models of scanners. This remains an area for potential future research and development.

This study has critical implications for researchers, clinicians and patients. For researchers, the developed deep learning model provides a reliable and efficient tool for musculoskeletal tissue segmentation, supporting further studies on musculoskeletal health and enhancing our understanding of conditions such as osteoporosis and sarcopenia. Future versions of this technique have the potential to benefit clinicians by providing an automated segmentation approach that is fast, accurate, and less dependent on manual input, enabling more efficient diagnostic processes and allowing for earlier detection of tissue loss or fat infiltration in the hip region. For patients, opportunistic utilization of the routine CT scans using our technique can provide precise quantification of tissue changes associated with ageing and chronic conditions, leading to more personalized and timely interventions that may reduce the risk of falls and fractures. Overall, these findings suggest that tissue quantification in this area offers a valuable, practical, and low‐cost tool that could be readily implemented in clinical practice. However, validation in larger cohorts through longitudinal studies that include both sexes is necessary to fully confirm its efficacy.

## Conflicts of Interest

The authors declare no conflicts of interest.

## Supporting information


**Figure S1** Schematic representation of the architecture used in this study.

**Figure S2** Schematic representation of the architecture used in this study.

**Figure S3** Correlation among area measures. A strongest correlation was observed between total bone marrow and MAT (*r* = 0.754), and between cortical bone and total bone marrow (*r* = −0.588).

**Figure S4** Correlation between the measurements for area and volume for both datasets.

**Data S1** Supporting information.

## Data Availability

Analysed data are available in this manuscript. Raw data are available on the MrOS website (access is subject to approval): https://mrosonline.ucsf.edu.
