# Supplementary figures and images for: Deep Learning Technique for Automatic Segmentation of Proximal Hip Musculoskeletal Tissues From CT Scan Images: A MrOS Study
Source: J Cachexia Sarcopenia Muscle. 2025 Feb 28;16(2):e13728. doi: 10.1002/jcsm.13728 (PMC11871091; doi:10.1002/jcsm.13728)

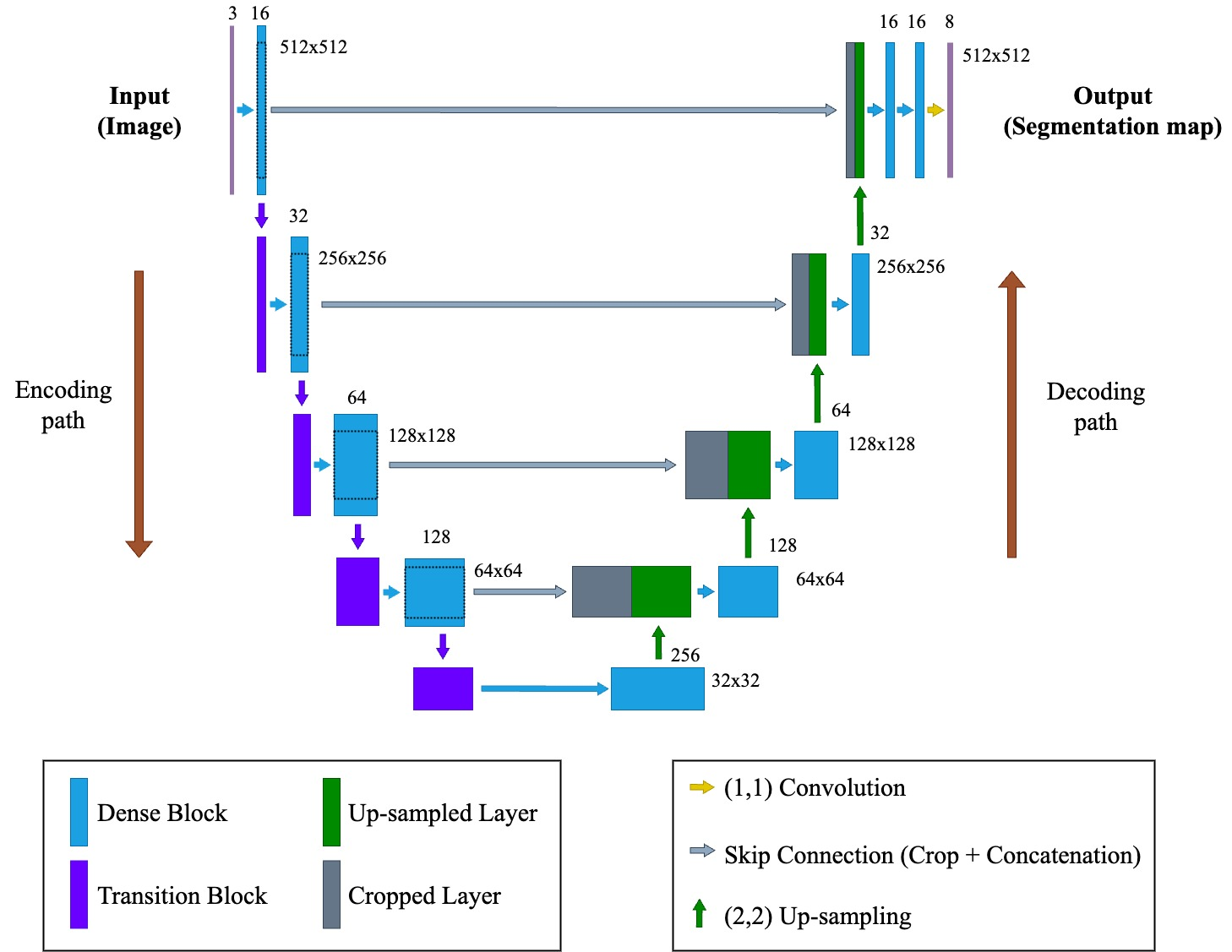

Supplement: Supplementary file 1 — Figure S1 Schematic representation of the architecture used in this study. [file JCSM-16-e13728-s003.tiff]

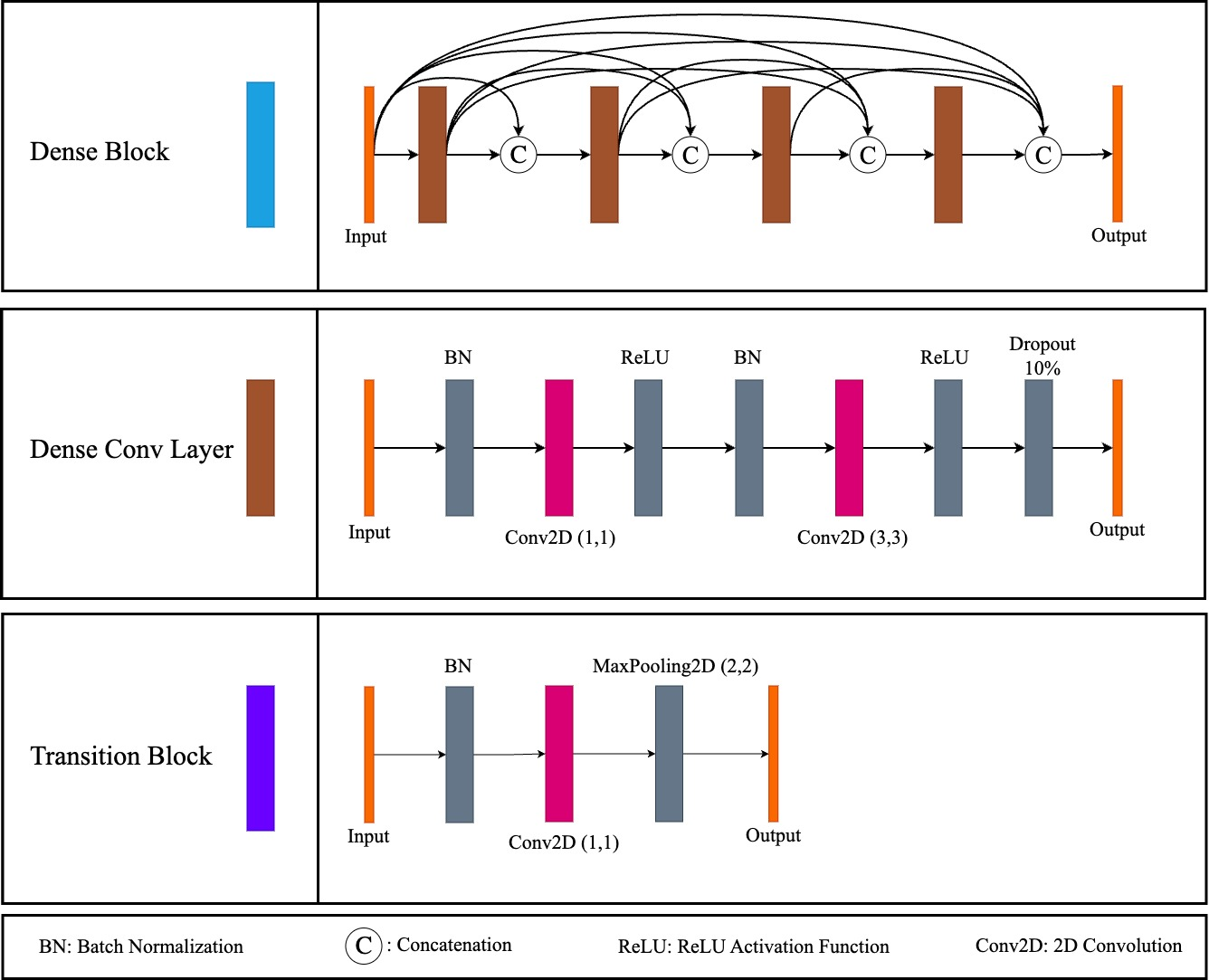

Supplement: Supplementary file 2 — Figure S2 Schematic representation of the architecture used in this study. [file JCSM-16-e13728-s002.tiff]

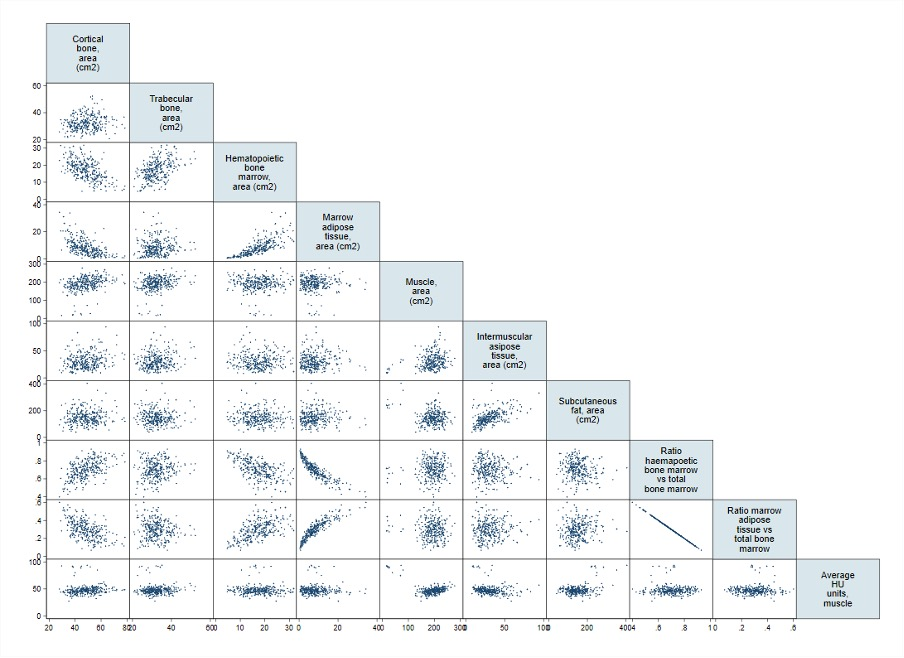

Supplement: Supplementary file 3 — Figure S3 Correlation among area measures. A strongest correlation was observed between total bone marrow and MAT (r = 0.754), and between cortical bone and total bone marrow (r = −0.588). [file JCSM-16-e13728-s005.tiff]

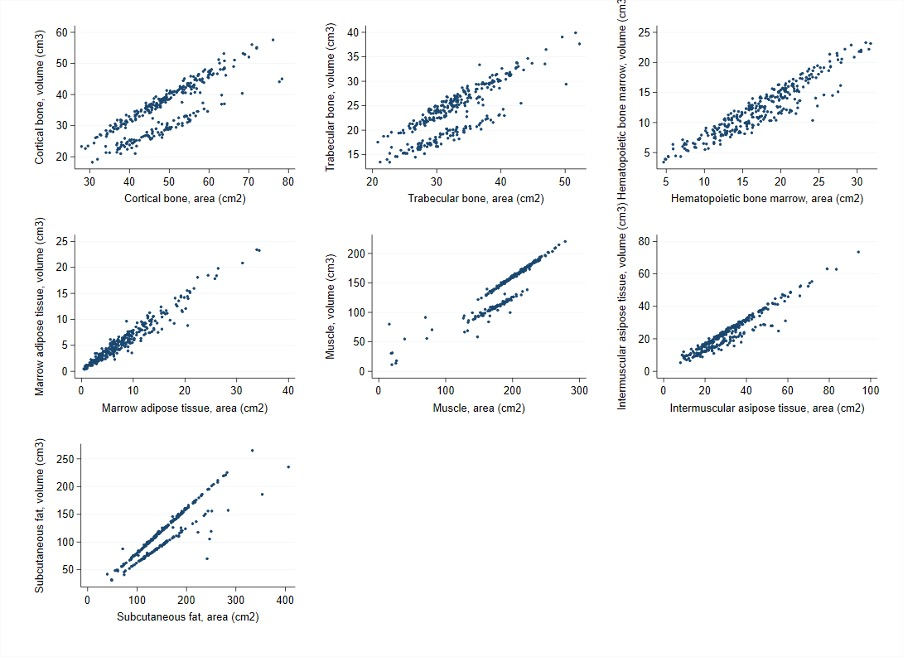

Supplement: Supplementary file 4 — Figure S4 Correlation between the measurements for area and volume for both datasets. [file JCSM-16-e13728-s001.tiff]
