# Supplementary material for: Deep Learning Technique for Automatic Segmentation of Proximal Hip Musculoskeletal Tissues From CT Scan Images: A MrOS Study
Source: J Cachexia Sarcopenia Muscle. 2025 Feb 28;16(2):e13728. doi: 10.1002/jcsm.13728 (PMC11871091; doi:10.1002/jcsm.13728)
Supplement: Supplementary file 5 — Data S1 Supporting information. [file JCSM-16-e13728-s004.docx]

S1. Avin, K.G. and R.N. Moorthi, *Bone is not alone: the effects of skeletal muscle dysfunction in chronic kidney disease.* Current osteoporosis reports, 2015. **13**: p. 173-179.

S2. Anderson, D.E., E. Quinn, E. Parker, B.T. Allaire, J.W. Muir, C.T. Rubin, . . . D.P. Kiel, *Associations of computed tomography-based trunk muscle size and density with balance and falls in older adults.* Journals of Gerontology Series A: Biomedical Sciences and Medical Sciences, 2016. **71**(6): p. 811-816.

S3. Hirschfeld, H., R. Kinsella, and G. Duque, *Osteosarcopenia: where bone, muscle, and fat collide.* Osteoporosis International, 2017. **28**: p. 2781-2790.

S4. Guerri, S., D. Mercatelli, M.P.A. Gómez, A. Napoli, G. Battista, G. Guglielmi, and A. Bazzocchi, *Quantitative imaging techniques for the assessment of osteoporosis and sarcopenia.* Quantitative imaging in medicine and surgery, 2018. **8**(1): p. 60.

S5. Byun, S.-E., S. Kim, K.-H. Kim, and Y.-C. Ha, *Psoas cross-sectional area as a predictor of mortality and a diagnostic tool for sarcopenia in hip fracture patients.* Journal of bone and mineral metabolism, 2019. **37**: p. 871-879.

S6. Lee, K., Y. Shin, J. Huh, Y.S. Sung, I.-S. Lee, K.-H. Yoon, and K.W. Kim, *Recent issues on body composition imaging for sarcopenia evaluation.* Korean journal of radiology, 2019. **20**(2): p. 205-217.

S7. Grau, V., A. Mewes, M. Alcaniz, R. Kikinis, and S.K. Warfield, *Improved watershed transform for medical image segmentation using prior information.* IEEE transactions on medical imaging, 2004. **23**(4): p. 447-458.

S8. Mishra, A.K., P.W. Fieguth, and D.A. Clausi, *Decoupled active contour (DAC) for boundary detection.* IEEE Transactions on Pattern Analysis and Machine Intelligence, 2010. **33**(2): p. 310-324.

S9. Yokota, F., Y. Otake, M. Takao, T. Ogawa, T. Okada, N. Sugano, and Y. Sato, *Automated muscle segmentation from CT images of the hip and thigh using a hierarchical multi-atlas method.* International journal of computer assisted radiology and surgery, 2018. **13**: p. 977-986.

S10. LeCun, Y., Y. Bengio, and G. Hinton, *Deep learning.* nature, 2015. **521**(7553): p. 436-444.

S11. Haque, H., M. Hashimoto, N. Uetake, and M. Jinzaki, *Semantic segmentation of thigh muscle using 2.5 d deep learning network trained with limited datasets.* arXiv preprint arXiv:1911.09249, 2019.

S12. Hemke, R., C.G. Buckless, A. Tsao, B. Wang, and M. Torriani, *Deep learning for automated segmentation of pelvic muscles, fat, and bone from CT studies for body composition assessment.* Skeletal radiology, 2020. **49**: p. 387-395.

S13. Nowak, S., A. Faron, J.A. Luetkens, H.L. Geißler, M. Praktiknjo, W. Block, . . . A.M. Sprinkart, *Fully automated segmentation of connective tissue compartments for CT-based body composition analysis: a deep learning approach.* Investigative radiology, 2020. **55**(6): p. 357-366.

S14. Blanc-Durand, P., J.-B. Schiratti, K. Schutte, P. Jehanno, P. Herent, F. Pigneur, . . . A. Luciani, *Abdominal musculature segmentation and surface prediction from CT using deep learning for sarcopenia assessment.* Diagnostic and Interventional Imaging, 2020. **101**(12): p. 789-794.

S15. Orwoll, E., J.B. Blank, E. Barrett-Connor, J. Cauley, S. Cummings, K. Ensrud, . . . L.M. Marshall, *Design and baseline characteristics of the osteoporotic fractures in men (MrOS) study—a large observational study of the determinants of fracture in older men.* Contemporary clinical trials, 2005. **26**(5): p. 569-585.

S16. Kanis, J.A., J.D. Adachi, C. Cooper, P. Clark, S.R. Cummings, M. Diaz-Curiel, . . . D. Pierroz, *Standardising the descriptive epidemiology of osteoporosis: recommendations from the Epidemiology and Quality of Life Working Group of IOF.* Osteoporosis International, 2013. **24**: p. 2763-2764.

S17. Khan, S., R. Warkhedkar, and A. Shyam, *Analysis of Hounsfield unit of human bones for strength evaluation.* Procedia materials science, 2014. **6**: p. 512-519.

S18. Rutten, I.J., D.P. van Dijk, R.F. Kruitwagen, R.G. Beets‐Tan, S.W. Olde Damink, and T. Van Gorp, *Loss of skeletal muscle during neoadjuvant chemotherapy is related to decreased survival in ovarian cancer patients.* Journal of cachexia, sarcopenia and muscle, 2016. **7**(4): p. 458-466.

S19. Zhang, Z. and M. Sabuncu, *Generalized cross entropy loss for training deep neural networks with noisy labels.* Advances in neural information processing systems, 2018. **31**.

S20. Dice, L.R., *Measures of the amount of ecologic association between species.* Ecology, 1945. **26**(3): p. 297-302.

S21. Heimann, T., B. Van Ginneken, M.A. Styner, Y. Arzhaeva, V. Aurich, C. Bauer, . . . G. Bekes, *Comparison and evaluation of methods for liver segmentation from CT datasets.* IEEE transactions on medical imaging, 2009. **28**(8): p. 1251-1265.

S22. Brady, S.L., A.T. Trout, E. Somasundaram, C.G. Anton, Y. Li, and J.R. Dillman, *Improving image quality and reducing radiation dose for pediatric CT by using deep learning reconstruction.* Radiology, 2021. **298**(1): p. 180-188.

S23. Mayo-Smith, W.W., A.K. Hara, M. Mahesh, D.V. Sahani, and W. Pavlicek, *How I do it: managing radiation dose in CT.* Radiology, 2014. **273**(3): p. 657-672.

S24. Bridge, C.P., M. Rosenthal, B. Wright, G. Kotecha, F. Fintelmann, F. Troschel, . . . A. Babic. *Fully-automated analysis of body composition from CT in cancer patients using convolutional neural networks*. in *OR 2.0 Context-Aware Operating Theaters, Computer Assisted Robotic Endoscopy, Clinical Image-Based Procedures, and Skin Image Analysis: First International Workshop, OR 2.0 2018, 5th International Workshop, CARE 2018, 7th International Workshop, CLIP 2018, Third International Workshop, ISIC 2018, Held in Conjunction with MICCAI 2018, Granada, Spain, September 16 and 20, 2018, Proceedings 5*. 2018. Springer.

S25. Dabiri, S., K. Popuri, E.M.C. Feliciano, B.J. Caan, V.E. Baracos, and M.F. Beg, *Muscle segmentation in axial computed tomography (CT) images at the lumbar (L3) and thoracic (T4) levels for body composition analysis.* Computerized Medical Imaging and Graphics, 2019. **75**: p. 47-55.

S26. Miao, S., H. Jia, K. Cheng, X. Hu, J. Li, W. Huang, and R. Wang, *Deep learning radiomics under multimodality explore association between muscle/fat and metastasis and survival in breast cancer patients.* Brief Bioinform, 2022. **23**(6).

S27. Yang, Q., X. Yu, H.H. Lee, Y. Tang, S. Bao, K.S. Gravenstein, . . . B.A. Landman. *Quantification of muscle, bones, and fat on single slice thigh CT*. in *Medical Imaging 2022: Image Processing*. 2022. SPIE.

S28. Yang, Q., X. Yu, H.H. Lee, Y. Tang, S. Bao, K.S. Gravenstein, . . . B.A. Landman, *Label efficient segmentation of single slice thigh CT with two-stage pseudo labels.* Journal of Medical Imaging, 2022. **9**(5): p. 052405-052405.

S29. Iwasa, M., M. Takao, M. Soufi, K. Uemura, Y. Otake, H. Hamada, . . . S. Okada, *Artificial intelligence-based volumetric analysis of muscle atrophy and fatty degeneration in patients with hip osteoarthritis and its correlation with health-related quality of life.* International Journal of Computer Assisted Radiology and Surgery, 2023. **18**(1): p. 71-78.

S30. Neumann, D.A., *Kinesiology of the hip: a focus on muscular actions.* Journal of Orthopaedic & Sports Physical Therapy, 2010. **40**(2): p. 82-94.

S31. Orwoll, E.S. and R.F. Klein, *Osteoporosis in men: epidemiology, pathophysiology, and clinical characterization*, in *Osteoporosis*. 2001, Elsevier. p. 103-149.

S32. Wang, L., L. Yin, Y. Zhao, Y. Su, W. Sun, Y. Liu, . . . X. Cheng, *Muscle density discriminates hip fracture better than computed tomography X‐ray absorptiometry hip areal bone mineral density.* Journal of Cachexia, Sarcopenia and Muscle, 2020. **11**(6): p. 1799-1812.

S33. Cree, M.G., D. Paddon-Jones, B.R. Newcomer, O. Ronsen, A. Aarsland, R.R. Wolfe, and A. Ferrando, *Twenty-eight-day bed rest with hypercortisolemia induces peripheral insulin resistance and increases intramuscular triglycerides.* Metabolism, 2010. **59**(5): p. 703-710.

S34. Wronski, T., E. Morey-Holton, and W. Jee, *Skeletal alterations in rats during space flight.* Advances in Space Research, 1981. **1**(14): p. 135-140.

S35. Murphy, R.A., I. Reinders, T.C. Register, H.N. Ayonayon, A.B. Newman, S. Satterfield, . . . T.B. Harris, *Associations of BMI and adipose tissue area and density with incident mobility limitation and poor performance in older adults.* The American journal of clinical nutrition, 2014. **99**(5): p. 1059-1065.

S36. Lim, J.P., M.S. Chong, L. Tay, Y.X. Yang, B.P. Leung, A. Yeo, . . . W.S. Lim, *Inter-muscular adipose tissue is associated with adipose tissue inflammation and poorer functional performance in central adiposity.* Arch Gerontol Geriatr, 2019. **81**: p. 1-7.

S37. Bhasin, S., T.G. Travison, T.M. Manini, S. Patel, K.M. Pencina, R.A. Fielding, . . . C. Cooper, *Sarcopenia definition: the position statements of the sarcopenia definition and outcomes consortium.* Journal of the American Geriatrics Society, 2020. **68**(7): p. 1410-1418.

S38. Byun, S.-E., S. Kim, K.-H. Kim, and Y.-C. Ha, Psoas cross-sectional area as a predictor of mortality and a diagnostic tool for sarcopenia in hip fracture patients. Journal of bone and mineral metabolism, 2019. 37: p. 871-879.
